# Potential Hazards Associated with Raw Donkey Milk Consumption: A Review

**DOI:** 10.1155/2019/5782974

**Published:** 2019-06-02

**Authors:** F. Conte, A. Panebianco

**Affiliations:** Department of Veterinary Sciences, University of Messina, 98168 Messina, Italy

## Abstract

Donkey milk can be used as a substitute for infants and children who suffer from cow milk proteins intolerance and multiple food hypersensitivity. Up to date, this is one of the main reasons why donkey milk has become a substantial area for reasearch, with an increase over the the last fifteen years. In donkey milk chain, risk analysis should be the object of particular attention because children are the main consumers of this food. In fact, this process is one of the main tool to achieve a high level of protection of human health and life; thus, the most important safety hazards should be monitored in order to attain this goal. This review focuses on the main hazards possibly present in raw donkey milk, including bacteria, fungal toxins, parasites, and chemical pollutants. Literature data have been considered, including some information that is not provided in the international literature. In the authors' opinion, the current scientific knowledge should be improved, with the aim of allowing a suitable risk assessment along the whole donkey milk chain. However, in the meantime, the competent authorithies must carry out more stringent official controls, with particular attention given to the level of primary production. The issue of a traceability system in donkey milk chain should be considered of paramount importance.

## 1. Introduction

Cow, goat and sheep's milk account for the majority of the global production; buffalo milk production is in second place worldwide [1]. Bovine milk represents over 80% of the world's milk production and is a major source of essential nutrients for growth, development, and maintenance of human health [2].

Minor dairy animal species are nutritionally and economically important in several countries; despite this, donkey, Bactrian camel, reindeer, musk ox, llama, moose, yak, and alpaca have been regarded as underutilized milk-producing animals and are defined as “species with underexploited potential for contributing to food security, health and nutrition.” [3]

Donkey milk is used as breast-milk substitute in European small-scale farms that choose diversified production. Donkeys, horses, and yaks production accounts for less than 0.1%, compared to all species, including cattle, but no world-specific statistics are available. Nevertheless, at present, the consumers' interest in donkey milk is increasing, and this product is gaining importance and international acceptance. This food is regarded as a “niche business” with high commercial value [1]; nowdays, it is used in maternity hospitals for the feeding of infants, for example in Italy. Furthermore, until the beginning of the 20th century, it was meant for the feeding of orphan infants, unhealthy children, and the elderly. It can be considered as an alternative ingredient in the “solid food-based diet” or after the first year of life in sensitive infants [4]. The use of donkey milk was considered an important solution for the treatment of infants with multiple food intolerance; in this case, it needs to be complemented with medium-chain triglycerides to reach the daily caloric intake recommended during the growth recovery phase in distrophic patients [5]. Throughout time, it was confirmed that donkey milk feeding can offer an important solution for the treatment of the most complicated cases of multiple food intolerance in young children affected by cow milk allergy [6, 7]. High content of *ω*-3 fatty acids supports the use of donkey milk as an effective functional food, in the prevention of cardiovascular diseases, and chronic inflammatory processes; in addition, the high percentage of medium and short-chain fatty acids potentiates the antioxidant properties of this milk. Both colostrum and milk from donkey may be useful in the treatment of human immune-related diseases. It may be helpful in the prevention of atherosclerosis, in view of strong vasodilatory and antimicrobial properties. In fact, pathogens and/or their products may play a proatherogenic role [8–10].

In Italy, donkey milk is configured as “pharmafood” for its nutritional, nutraceutical, and functional properties [11]. The raw milk for human consumption can be sold directly at farms; it is also pasteurized (rarely, UHT-treated or freeze-dried), packed, and sold in shops, pharmacies, or it is sold online [12]. It is not always or easily available on the market and domestic milk freezing of donkey milk is a common practice [13].

The traditional use of donkey milk would be the reason why donkeys farms were set up in Italy, France, Belgium, Switzerland, and Germany at the beginning of the 20th century [4]. In Italy, new farms were built in several regions [12], and Sicily was defined a “leading producer” of donkey milk [14].

In some geographical areas, raw donkey milk consumption is not unusual, as is the case of cow milk, in order to avoid thermal degradation of the valuable substances. This trend holds a risk for the consumer, due to the possible occurence of human pathogenic microorganisms in raw milk [15].

As mentioned above, raw milk from donkey is sold at farms and only refrigerated between 0 and 4°C. Given the current increase of donkey milk demand, it could be considered as a potential health hazard source [16]; in fact, contamination caused by human pathogens would seem feasible. It is, therefore, strongly recommended to heat milk before consumption, especially by infants, with the purpose of better ensuring their health protection.

“The pursuit of a high level of protection of human life and health” is one of the fundamental objectives of European food hygiene rules. “An integrated approach is necessary to ensure food safety from the place of primary production up to, and including, placing on the market or for export.” [17]

One of the main tools for protecting human health is “risk analysis” because it “provides a systematic methodology for the determination of effective, proportionate and targeted measures or other actions to protect health.” [18]

Regulation 2017/625/EU on “Official Controls” strictly relates to the “risk analysis”; in fact, it states that official controls should be performed regularly, on a risk basis and with appropriate frequency; and besides, the latter is also defined on a risk basis [19].

In donkey milk chain, special attention should be paid to the “risk analysis,” in view of its principal destination, namely, the infants who are affected by multiple food intolerance or cow milk allergy.

Raw donkey milk assessment should preferably focus on the potential and most relevant hazards throughout the milk chain. For this purpose, the presence of biological (bacteria, parasites, and virus) and chemical hazards should primarily be assessed.

The aim of this review is to present scientifically sound information on potential hazards along the donkey milk chain. For this reason, data from the available literature on various topics will be reported, including some studies which were not provided in the international research networks. This review paper should be seen as a contribution toward the better understanding of the current risk assessment in the donkey milk chain.

## 2. Microbiological Hazards

### 2.1. Foodborne Pathogens

Microbial contamination of milk can originate from the interior and the exterior of the udder, from handling practices, and the storage equipment [20].

A primary route of pathogen transmission in milk is fecal contamination during milking [20].

Intrinsic contamination of milk may result from systemic disease in the animal or localized infection such as mastitis. Subclinical mastitis are those for which no visible changes occur in the appearance of milk or the udder; if this milk is poured into the bulk tank, it enters the food chain and can be hazardous to consumer health.

It seems that raw donkey milk generally does not harbor foodborne pathogens, thanks to its antimicrobial properties [21]; this statement can be only partially shared.

Bacterial microflora isolated from donkey milk, mainly in Europe, can be briefly summarized as follows:* Bacillus (B.) cereus, Campylobacter *spp., coliforms,* Cronobacter (Cr.) sakazakii *(formerly* Enterobacter sakazakii*)*, Enterobacter (En.) cloacae, En. agglomerans, Escherichia coli*,* Escherichia *(*E.*)* hermannii*,* Listeria* spp.,* Pseudomonas (Ps) aeruginosa*,* Staphylococcus (S.) aureus S. chromogenes, S. intermedius*,* S. sciuri, S. warneri*,* S. xylosus*,* Streptococcus (Str.) hycus*,* Str. epidermidis, Str. equi*,* Str. equisimilis*,* Str. intermedius*,* Str. zooepidemicus*,* Str. dysgalactiae* [22]. More details are shown in Table 1.

*En. sakazakii (*at present* Cr. sakazakii*) was isolated for the first time from 2 raw donkey milk samples. Two* En*.* cloacae* and one* En. agglomerans* strains, from different samples, were also recovered. In the case report, it was stressed that for a proper risk assessment for* Cr*.* sakazakii* a good knowledge on its occurrence in foods is necessary, especially those intended for infants. Furthermore, the risk assessment could not be developed without utilizing knowledge from epidemiological studies. The authors have expressed their concern about the possibility that donkey milk could become a high-risk food [23].

The isolation of 176* Staphylococcus* spp. strains from donkey milk samples was reported; coagulase positive strains were recovered; 27 out of 30 specimens were identified as* S. aureus*, 1 as* S. intermedius*, and 2 as* S. chromogenes; *a total of 146 coagulase negative strains were found [24].

Coagulase-positive staphylococci were found in the raw donkey milk, with an average count of 1.7 × 10^2^ cfu/ml [13].

*Bacillus cereus* was detected in 3 bulk milk samples (maximum concentration: 1.2 x 10^3^ cfu/ml), in 3 individual milk samples (10, 20, and 60 cfu/ml, respectively), in the milk filter (5 cfu/cm^2^), in the soil (maximum concentration: 1.5 x 10^3^ cfu/g), on the hands and gloves of two milkers, and on animal hide (from 1 to 3 cfu/cm^2^), but no spores were detected. A total of 8* Bacillus cereus sensu strictu* strains were analyzed for diarrhoic toxin, and 6 strains were enterotoxins producing [25]. In further donkey milk samples in Italy,* B. cereus* was isolated by other researchers [26].

Alberghini et al. [27] identified 1* Campylobacter coli* strain by multiplex polymerase chain reaction (PCR) in donkey milk from a farm in north Italy; furthermore, for one* E.coli* O157 strain, the authors indicated the presence of verocitotoxins encoding genes [27].

*E. coli* strains isolated in Iran from raw donkey milk were Shiga toxin-producing (STEC) serotypes; particularly, four were O157 and one was O111 serotypes, respectively. Twelve different virulence genes were isolated from these strains. The study introduced donkeys as reservoirs of* E. coli* O157 for the first time, as well as camels and buffaloes. [28].

Mottola et al. [29] showed that out of 90 samples of donkey milk, one (1.11%) contained* E. coli* O157 harboring the Shiga-like- toxins (SLT-I and SLT-II); in one sample (1.11%)* Campylobacter (C.) coli *was recovered. The authors stated that the isolation of* C. coli* and* E. coli* O157 in donkey milk aroused a great public health issue. The presence of these bacterial species was attributed to several factors, such as farm size, number of animals on the farm, and hygiene and management practices. Generally, the detection of Enterobacteriaceae in donkey milk shows the importance to improve the hygienic practices on farm level [30].

### 2.2. Mastitis Agents

Some bacterial species, recovered from donkey milk samples, may act as pathogens for mammary gland, resulting into an inflammation.

Mastitis is a well-known problem for dairy farms. Udder inflammation can be caused by a large variety of bacteria including* S. aureus*, some coliforms, and Brucella (for some EU countries); they are frequently found in infected animals and can be transmitted to humans through milk [31].

Only limited data are available in the literature concerning the incidence of mastitis in donkey, as well as in mare.

Three cases of subclinical udder inflammation from donkeys reared in Sicily were described; quantitative, cytological, and bacteriological evaluations were referred. Somatic cells count was very high in milk samples (2.639.000, 3.897.000, and 4.543.000 cells/ml, respectively). One* Staphylococcus aureus* strain and one* Pseudomonas* sp. strain were isolated from two different milk samples; no bacterial specimen was identified from the third milk sample. Cytological pictures allowed a diagnosis of subclinical mastitis. Because of the rarity of the observed findings and the lack of references on the topic, the case reports were considered very interesting [32].

Pilla et al. [24] described several cases of mastitis in donkeys caused by* S. aureus *(5 strains),* Str. equi *(2 strains),* Str. equisimilis *(1 strain),* Str. acidominimus* (1 strain), and coagulase negative staphylococci (1 strain). No* S. aureus* isolate carried the genes coding for any enterotoxin, toxic-shock syndrome toxin, or antibiotic resistance [24].

*S. aureus* and* Str. equi* subsp.* zooepidemicus, *as mastitic agents, were isolated in one and in two donkey milk samples, respectively. The isolation concerned the individual milk samples collected during the second lactation period; on the contrary, the samples examined during the first lactation period were negative for mastitic agents.

The authors stated that the isolation of* S. aureus* in milk emphasised the importance of preventing contamination in the primary production to ensure the achievement of the objectives reported in the relevant community legislation [33].

Several situations can increase the risk for mastitis, including postmilking teat disinfection, poor hygiene of milking equipment, barn type, drinking water quality, etc.

### 2.3. Antimicrobial-Resistant Bacteria in Donkey Milk

The development of bacterial resistance to antimicrobial agents poses a serious threat to human health. Raw milk may be a source of bacteria (primary or opportunistic pathogens) that are resistant to antimicrobials. Transfer of resistance affects the emergence and selection of multidrug-resistant foodborne pathogens. Raw milk may be a source of antimicrobial-resistant bacteria, depending on the reservoir of bacteria in the farm and in the animals environment [20, 34].

In raw milk from donkey, antibiotic resistance (AR) was described in two* En. sakazakii *(at present* Cr*.* sakazakii*) strains from 2 samples; bacterial strains were assessed for antibiotic susceptibility to 33 molecules. Both strains were susceptible to aminoglycosides; one strain was sensitive to ciprofloxacin, oxolinic acid, and framicetin. The resistance to macrolides, novobiocins, penicillins, cephazolin, ceftazidime, cephotaxime, and cephalotin has been emphasised. In fact, some molecules, such as nalidixic acid, ciprofloxacin, and oxolinic acid, are commonly used to treat infected human patients [23].

Four coagulase positive staphylococci and ten coagulase negative strains were isolated from donkey milk samples; the strains were methicillin (METH) resistant; 7 coagulase positive and 25 coagulase negative strains were oxacillin (OXA) resistant. Five coagulase negative strains, susceptible to METH and OXA, were positive for mecA gene. According to the authors, these strains could play a significant role in the colonization of various animals; the latter could become carriers of resistance determinants, with subsequent dissemination and trasmission to pathogens. Humans could acquire the saprophytic and pathogenic flora by consuming donkey milk [35].

In a further study,* S. aureus*,* Str. equi*,* Str. equisimilis*, and* Str. acidominimus, *recovered from donkeys affected by mastitis, exhibited no resistance to the tested antibiotics. Indeed, all bacteria species were sensitive to the antibiotics used in veterinary practice (*β*-lactams or methicillin, aminoglycosides, macrolides, vancomycin, and lincosamides or tetracyclines) [24].

*E. coli* strains were isolated in Iran from raw donkey milk;* E. coli* O157 was the most prevalent serotype in milk samples. One O157 serotype showed the highest AR to penicillin and enrofloxacin [28].

The potential hazards related to antibiotic resistance should also concern donkey milk; the literature data on this topic are extremely scarce.

## 3. Other Potential Biological Hazards

Risk occurrence can only be hypotesized for some microbiological hazards; the literature data on the topic are reported below.

### 3.1. *Brucella* spp.

Brucellosis has been eradicated in many developed countries like Europe, Australia, Canada, Israel, Japan, and New Zealand. However, it is still endemic in most areas of the world, such as the Africa, Mediterranean, Middle East, parts of Asia, and Latin America. Nearly, all animal species are susceptible; the prevalence of brucellosis varies very widely in equine, bovine, caprine, ovine, and camelidae; humans have the least prevalence [36].

*Brucella melitensis* might infect equids, and further studies are required on the isolation of the organism and the role played by equids in the epidemiology of brucellosis.

It was shown that donkeys and horses are not a reservoir of brucellosis in some geografical areas in Mexico [37]. On the contrary, in Sudan, equines may be a reservoir of brucellosis and may also play an important role in the epidemiologic patterns of this disease in the sampled geographical area [38].

The low prevalence of intramammary infections in donkeys suggests that milk might be considered a safe food. Unfortunately, in some areas of the world (e.g., northeastern Nigeria), these animals are a potential source of* Brucella* infection, both for people living in close contact with donkeys [39] and through ingestion of unpasteurised milk [40].

### 3.2. *Mycobacterium* spp.

Isolation of* Mycobacterium (M)* spp. seems not to occur in donkey milk. Horses are considered very resistant to mycobacterial infections; similarly, it could be hypotesized for donkeys [41].

The incidence of tuberculosis (TB) in equids is extremely low, especially in countries with established control programs. In contrast to* M. bovis* that is known to affect a wide range of natural hosts, susceptibility to* M. tuberculosis* remains not well defined for many mammal species including horses and other mammals (e.g., donkeys, goats, and sheep) even after a direct or indirect contact with infected animals [42].

### 3.3. *Toxoplasma gondii*

Any warm-blooded animal including most pets, livestock, birds, and humans can become infected with* Toxoplasma (T.) gondii. *This protozoan has developed several potential routes of transmission within and between different host species. Infection occurs mainly within congenital and horizontal transmission; the latter may also occur through ingesting infectious oocysts from the environment or tissue cysts or tachyzoites contained in meat, offal of many different animals; raw or undercooked milk, and unwashed fruit and vegetables [43, 44].

Although the “frequency of occurrence” of* T. gondii* in raw milk seems to be unknown [15], the single-celled parasite was found in milk of several intermediate hosts, e.g., sheep, goat, donkey, camel, buffalo, and breast-fed infant whose mother acquired a primary infection with* T. gondii*. Any type of raw milk is a potential source of toxoplasmosis [43, 44].

Little is known about* T. gondii* infection in donkeys [43], and donkey milk contamination by* T. gondii* is not well documented. In the milk of pregnant Egyptian females, the antibodies against toxoplasmosis were detected by enzyme-linked immunosorbent assay (ELISA); milk from 15 out of 75 donkeys was positive in 7 samples, with a contamination rate of 46.3% [45].

Mancianti et al. [16] detected the parasite in Italian donkeys using molecular tools; the Nested-PCR technique showed that 3 out of 6 tested milk samples were contaminated.

In further studies, the effects of* T. gondii* on milk safety, yield, and quality in 18 sero-positive donkeys with parasitemia were investigated. The results of serological test showed 4 positivities (22.22%) for* T. gondii*, and each serological positive donkey presented parasitic DNA both in the blood and milk. In the light of preliminary results, the authors believe that* in vivo* studies are needed to assess more thoroughly the risk of transmission of* T. gondii* through donkey milk [46].

### 3.4. *Cryptosporidium *spp. and Microsporidia

Criptosporidia are parasites colonizing digestive and/or respiratory systems of birds, fish, reptiles, and mammals, including equines.

*Cryptosporidium* spp. are responsible for diarrhea in humans and animals. The infective oocysts from both are ubiquitous in the environment, and cryptosporidiosis can be acquired via the fecal-oral route directly from infected humans or animals or indirectly from food or water contaminated with the faeces of infected hosts.

The isolation of zoonotic parasites, including* Cryptosporidium* (*C.*) and Microsporidia, from fecal sample of donkeys and horses, has shown that these equids have the potential to transmit human-pathogenic parasites. This risk appears to be negligible, given the low prevalence of all parasitic taxa [47].

Nevertheless, the risk of human infection through donkey milk consumption should be considered.

Compared to cryptosporidiosis in horses, the knowledge in donkeys is poor, with particular reference to the identity of species that might affect these equids; till date, only two* Cryptosporidium* species (*C. parvum* and* C. muris*) have been identified.

*C. parvum, C. erinacei*, and* Cryptosporidium* horse genotypes were detected in horses and donkeys and have caused infection in humans [47].

Microsporidian species have been increasingly recognized as opportunistic pathogens in immunodeficient patients [48]. Fecal-oral routes, such as ingestion of contaminated water and food, are the major routes of microsporidia transmission which consist of 1300 named species [49].

Among the species that infect humans,* Enterocytozoon (Er.) bieneusi*, is the most prevalent agent of diarrhea pulmonary and hepatobiliary diseases.* Encephalitozoon (Ez.) intestinalis* has been considered the second most prevalent microsporidian species, causing gastrointestinal infections.* Ez. intestinalis* spores were detected in faeces from several mammals including donkey, cow, goat, and pig. These animals harbored the spores and disseminated them into the environment [48]. This parasite affects donkeys, causing infections in the gastrointestinal, ocular, genitourinary, and respiratory tracts [50].

*Ez. intestinalis, Ez. cuniculi* genotypes I and II, and* Er. bieneusi* (genotypes D, EbpA, G, and WL15), detected in horses and donkeys, have been shown to cause human infection [50, 51]; no relation was found with donkey milk consumption.

In China,* Er. bieneusi* was first genotyped in donkeys worldwide. These equids were considered a potential source of animal and human microsporidiosis [49].

On the basis of the above, donkey milk contamination by microsporidia and consequent human infection cannot be ruled out.

### 3.5. *Giardia *spp.

*Giardia (G.) intestinalis* is the only species known to cause illness in humans, it is distributed worldwide and it can infect many vertebrates. The main symptom of giardiasis is diarrhea and its transmission is mainly through ingestion of* Giardia* cysts in contaminated food or water. The majority of cases of human giardiasis are reported in developing countries [52].

Although* G. intestinalis* infections are known in humans and in a variety of animal species, there is little information on donkeys (*Equus asinus*). Zhang et al. [53] suggested that donkeys could be a source of giardiasis outbreaks in China.

The zoonotic potential of these parasites in donkeys should be elucidated.

### 3.6. Tick-Borne Pathogens

The literature data did not show the recovery of tick-borne causative agents in donkey milk.

Tick-borne encephalitis virus (TBEV) is regarded as one of the most common and potentially fatal zoonoses affecting human central nervous system. TBE is endemic in Central and Eastern Europe, and Russia; a wide geographical areas can be involved, as for example Alsace Lorraine and Scandinavia, or northeast China, and northern Japan [20, 54].

Goats, sheep, and cattle are important for the so-called alimentary TBE [55]. During viraemia, TBEV is excreted into the milk and can be ingested via consumption of raw milk or cheese made from raw milk (mainly from goat) [20, 54].

As in the case of small ruminants, in donkeys the virus might be considered a significant hazard when the animals are exposed to ticks carrying TBEV that could be transferred to milk.

*Coxiella (C.) burnetii* is considered the most representative tick-borne pathogen in dairy animals. It is the causal agent of Q-fever, a zoonosis with a worldwide distribution. Numerous animals can be infected by* C. burnetii*; among livestock, dairy cattle, sheep, and goats are the major reservoirs of this rickettsial bacterium and are more frequently related to the outbreaks of human Q-fever than other animal species. The zoonosis is essentially airborne; infection from commercial milk is unlikely because a thermal treatment is used. Raw milk or dairy products from unpasteurized milk may harbor virulent* C. burnetii *[56].

To the best of our knowledge, having considered the literature on the topic,* C. burnetii* was never found in donkey milk.

## 4. Chemical Hazards

Unacceptable amounts of chemicals, and their residues in milk supply, pose a potential threat to human health, particularly children, who are the primary consumers and whose sensitivity is potentially greater than that of adults [2].

Chemical hazards may be introduced into milk during production, dairy processing, or packaging. Veterinary drugs, heavy metals, radionuclides, mycotoxins, and pesticides, as chemical contaminants, can enter animal feeds and leave their residues in milk [57].

Maximum levels for several contaminants in food were set, but non-regulated chemicals are of particular concern. Although competent authorities have taken adequate measures to minimize the individual exposure to food contaminants, further steps need to be taken to minimize the health risks related to chemical food contamination [58]. This is particularly true for donkey milk; in fact, there are only few data available on the topic; moreover, regulations on maximum levels of some contaminants in equids milk are lacking.

### 4.1. Potentially Toxic Trace Elements

Scientific reports on the levels of heavy metals in milk of several animal species are available from different geographical areas; on the contrary, there is only very limited information on trace elements in donkey milk.

The concentrations of nontoxic iron (Fe), zinc (Zn), chromium (Cr), copper (Cu), selenium (Se), and manganese (Mn), and potentially toxic elements arsenic (As), lead (Pb), cadmium (Cd), mercury (Hg), nickel (Ni), and antimony (Sb) in donkey milk, forage, and feed samples from three Italian farms (Sicily, Calabria, and Emilia Romagna) were reported.

Donkey milk would appear a good source of selenium for both adults and children; furthermore, the majority of milk samples did not reveal dangerous residue levels for human health. Cd concentrations were nearly similar to the values for cow milk from Calabria and Sicily [59]. Pb levels in some samples were higher than the European maximum level (0.02 mg/kg) [60]. Finally, it was underlined the need for further study on the quality and safety of donkey milk, providing more consistent data, in order to rule out any toxicological risks for human health [59].

On the basis of what is stated in Regulation 1881/2006/EC [60], the maximum limit of Pb fixed for “raw milk,” as defined in the Regulation 853/2004/EC [61], can be applied to the secretion of the mammary gland of farm animals. Consequently, the limit shall also apply to donkey milk.

In further studies on toxic metals, the concentrations of titanium (Ti) vanadium (V), As, rubidium (Rb), strontium (Sr), molybdenum (Mo), Cd, cesium (Cs), and Pb were assessed in donkey milk and blood serum from a dairy farm in the north of Italy.

The authors reported that As, Cd, and Pb concentrations were lower than those referred by other authors [59]. When compared with published data on human milk, donkey milk generally showed similar or lower concentrations of V and Mo, higher values of Ti and Sr, and lower values of Cs and Rb. Compared with cow milk, donkey milk revealed similar or lower levels of Ti, lower V, Mo,Rb, and Cs levels, and higher leveles of Sr [62].

Paksoy et al. [63] evaluated the concentrations of essential elements and heavy metals in milk of dairy donkeys, goats, and sheep in Turkey. Regarding heavy metals, the authors stated that Ni, Cd V and barium (Ba) concentrations (expressed in *μ*g/L) in donkey milk were lower than the detection limit (1 mg/L). The results of the study showed a low health risk of human exposure to heavy metals through milk consumption [64].

### 4.2. Pesticides

Milk-producing animals accumulate pesticides residues through contaminated feed, grass/hay and by air; donkey milk could be contaminated in the same way.

Current literature on pesticides in donkey milk is very scanty. The first report, in 2010, concerns the evaluation of organochlorine pesticides (OCPs) and polychlorinated biphenyls (PCBs) levels in donkey milk samples from three farms in Sicily [65].

The examined OCPs were 4,4′-DDE (dichlorodiphenyldichloroethylene), aldrin, and dieldrin; their amounts were always below the limits fixed by law for cow milk. Seven PCB target congeners were also assessed; furthermore, 12 Dioxin-Like PCBs (DL PCBs) were evaluated.

PCBs residues were observed in 80% of samples from the first farm, in 60% of samples from the second farm, and in 80% of samples from the last farm. Their sum was always below the limit of 100 ng/g of fat fixed for cow milk. Levels of six PCB congeners were lower than their quantification limit; in any case, the results were lower than the legal limit set for cow milk (3 pg/g fat) [65].

In a further study, donkey milk, forage, and feed samples were also examined; OCPs and PCBs amounts in donkey milk samples were the same as those reported by the authors in 2010 [65].

The study showed very low pesticides leveles in forage and feed samples from different farms. The amount of contaminants in donkey milk samples were considered similar to those found in other types of milk, as shown in the literature data. According to the authors [66], estimated daily intake (EDI) values suggested that the consumption of donkey milk will not present a health risk to consumers. As a consequence, the results did not cause any concern; despite this, children fed donkey milk are inevitably exposed to the examined pollutants [66].

### 4.3. Aflatoxins

Mycotoxins are natural contaminants produced by a range of fungal species (mainly* Aspergillus, Penicillium, Fusarium, Alternaria, *and* Claviceps* spp.) during plant growth in the field, harvesting, storage or feed processing. Mycotoxins detection in milk and dairy products mainly concerns aflatoxins (AFs), ochratoxin A (OTA), zearalenone (ZEN) and its metabolites, fumonisin (FUM), cyclopiazonic acid (CPA), sterigmatocystin (STC), and patulin (PAT) [19]. AFs are considered the most important for dietary exposure from dairy products and, subsequently, the only mycotoxins for which maximum limits have been established for milk and its products [31].

AFs are among the well known and widely investigated groups of mycotoxins which can be found as contaminants in food and feed worldwide. As it is well known, AFB1 and AFB2, after ingestion, are metabolized by the liver into their hydroxylated metabolites M1 (AFM1) and M2 (AFM2), which can be excreted in urine and feces, transferred to milk, and to a lesser extent, to meat. AFM1 is excreted into the milk of both lactating humans and animals after ingestion of contaminated food and feed, respectively [1, 67].

The presence of AFM1 in milk and dairy products worldwide has been known for a long time; nevertheless, milk and dairy products contamination is a relevant problem [68]; AFM1 in milk and dairy products could pose a risk to humans as well as animals' health. The presence of AFM1 in milk and dairy products is an important issue, relating to children and infants, who are more susceptible than adults [68].

Numerous studies on AFM1 in raw and heat-treated milk from several species have been presented [69]; literature data on AFM1 concentrations in sheep and goat milk are scarce in comparison to cow milk [67].

Recently, Iqbal et al. [69] provided an interesting review on different topics: the occurrence of AFM1 in milk and dairy products from many areas of the world; toxin stability during processing; strategies for its reduction; regulations; latest developments in detection methodologies; and future challenges [68].

Today, the available scientific data on AFM1 in donkey milk are lacking, especially when compared to the information on other dairy animals.

To the best of our knowledge, the first recovery of AFM1 in raw donkey milk was described in Sicily; levels lower than 5 ppt were found in bulk milk samples from Ragusano breed donkeys [70].

In Serbia, 5 raw donkey milk samples from small farms were assessed. The authors observed that data about AFM1 occurrence in donkey milk were very limited [71].

In donkey samples from Croatia, the AFM1 levels were comparable to the concentrations found in the milk of other species [67].

In Greece, no AFM1 residues were detected in 90 bulk donkey milk samples. Thirty-six samples were subsequently collected over a one-year period from 12 donkey farms across the same country. The toxin was found in 5 out of 36 samples (13.9%) [72]; concentrations were always lower than the EU maximum levels (50 ng/kg) [60, 72].

The authors reported that AFM1 levels in donkey milk were lower than the amount assessed in other milk types; this difference could be due to the feed and pasture type used for donkey feeding and also to the very low carryover of AFB1 to AFM1 that was reported in donkey. Furthermore, these animals are mainly fed with oats and barley that are not frequently carriers of AFB1, especially in Balkan and Mediterranean countries. The authors stressed that different techniques of analysis, sampling periods, and locations among studies on AFM1 levels in donkey milk, however, did not allow plausible comparison of data [72].

Only one study investigated the AFM1 carryover from feed to donkey milk; the carryover was clearly influenced by animal species; in particular, in monogastric animals, such us donkeys, it seems to be lower than that found in ruminants. According to the authors, further studies would be useful on this topic, and aflatoxins content in donkey milk should be taken into serious consideration [1].

The EU maximum limit that was set for AFM1 in raw milk shall also apply to secretion of the mammary gland of female farm animals and, therefore, to donkey.

### 4.4. Ivermectin

Different classes of drugs administred to horses and ruminants are also given to donkeys without dosage optimization, and determination of pharmacokinetic and pharmacodynamic properties. Because of the lack of registered drugs for donkeys, anthelmintics licensed for horses or ruminants are used in the treatment of parasitic infections in these equids [73].

Anthelmintic drugs have been shown to be effective for the control of parasitic infection in donkeys at doses determined for horses. The studies on the pharmacokinetics of some anthelmintics in donkeys support their use at these dosages, despite the apparent differences in absorption and elimination [74].

Ivermectin (IVM), a macrocyclic disaccharide anthelmintic agent, with broad-spectrum antiparasitic action, is used to control internal and external parasites in bovine, swine, and equides, including donkey [63]. Commission Implementing Regulation 418/2014/EU [75] set out the Maximum Residue Limits (MRLs) for IVM for all mammalian food producing species, applicable to muscle, fat, liver, and kidney. It is not for use in animals from which milk is produced for human consumption [75]; therefore, donkey milk should also not be used.

IVM is not licensed for use in lactating species in the EU, due to its persistent excretion in milk and its lipid-soluble character, which facilitates the absorption in humans after oral administration [63, 76].

As regards the excretion of macrocyclic lactones in milk, various investigations were carried out on dairy animals, including cattle, sheep, goat, buffalo, and camel; no information is available for donkey [73].

The presence of unknown or uncontrolled IVM residues in milk may cause potential risk to consumers' health. Producers must be informed about possible risks related to the use of this drug in lactating animals, and advice concerning this matter should be given to the veterinarians [63, 76].

## 5. Conclusions

In European legislation, it is clearly stated that “In order for there to be confidence in the scientific basis for food law, risk assessments should be undertaken in an independent, objective and transparent manner, on the basis of the available scientific information and data” [18].

In view of this statement, the present review should be seen as a deepening of information and data concerning some topics related to donkey milk chain. Further research and literature information would allow to carry out a proper risk assessment.

In the meantime, we can not set aside the fact that donkey milk is primarly intended for use in susceptible group of consumers (i.e., babies and infants); in view of this destination, it is necessary to pay special attention to the security of young consumers and provide stricter official controls. The latter must be applied mostly to records keeping in the primary production industry, as provided by Regulation 852/2004/EC [17].

Finally, an important issue is represented by an integral traceability system in donkey milk chain management; in fact, traceability can be considered as a tool to comply with the present legislation and to meet the food safety and quality requirements.

An effective traceability system will protect donkey milk against fraud or commercial disputes [85] as well as milk safety.

## Figures and Tables

**Table 1:** Microorganisms isolated from donkey milk.

Bacterial species	Microbial load [cfu/ml]	Country	References

*E. coli* *S. aureus*	10	Italy	Conte et al., 2002 [77]

*E. coli*	2.7x10^10^	Italy	Conte et al., 2003 [78]
*Staphylococcus *sp.	2x10^2^ 10		
*Ps. aeruginosa,* *Str. zooepidemicus,* *Str. intermedius,* *S. hyicus*	10		

*S. aureus, S. intermedius,* *Str. dysgalactiae,* *Str. epidermidis*	10	Italy	Conte et al., 2005[14]

*En. sakazakii* ^§^ *En. cloacae* *En. agglomerans*	n.d. ^§§^	Italy	Conte and Passantino, 2008 [23]

*S. aureus* *Fungi*	10^1^ 0.69∗	China	Zhang et al., 2008 [79]

*Staphylococcus *spp. *S. aureus, S. intermedius, S. chromogenes*	n.d. ^§^	Italy	Naccari et al., 2009 [35]

*S. aureus, Str. equi,* *Str. equisimilis,* *Str. acidominimus*		Italy	Pilla, 2010 [24]

*S. aureus* *E. coli* *Listeria* spp.	10 10^2^ n.d.	Italy	Colavita et al., 2010 [80]

*S. aureus*	27∗∗		Cascone et al., 2011 [70]

*S. aureus*	<10		Salerno et al., 2011 [81]

*B. cereus*	1.2 x 10^3^	Italy	Scatassa et al., 2011 [25]

*E. coli* (STEC serotypes)	n.d. ^§^	Iran	Momtaz et al., 2012 [28]

*S. aureus*	n.d. ^§^	Italy	Conte et al., 2012 [82]

*E. coli* O157 *Campylobacter coli*	<10	Italy	Albeghini et al., 2012 [27]

*B. cereus*	1.3x10^2^	Italy	Cavallarin et al., 2015[83]

*S. aureus* *Streptococcus equi subsp. zooepidemicus*	n.d.	Italy	Ragona et al., 2016 [33]

*Staphylococcus* spp. Yeasts and molds	1.6x10^3^ ∗∗ 4.9 x 10^3^∗∗	Greece and Cyprus	Malissiova et al., 2016 [84]

*B. cereus*	<10	Italy	Giribaldi et al., 2017 [26]

*E. coli* O157 *Campylobacter coli*	<10	Italy	Mottola et al., 2018 [29]

^§^At present *Cr. sakazakii*;  ^§§^ n.d.=not determined; ∗log cfu/ml; ∗∗m.v.=maximum value.

## References

[1] Tozzi B., Liponi G. B., Meucci V. (2016). Aflatoxins M1 and M2 in the milk of donkeys fed with naturally contaminated diet. *Dairy Science and Technology*.

[2] Wochner K. F., Becker-Algeri T. A., Colla E., Badiale-Furlong E., Drunkler D. A. (2018). The action of probiotic microorganisms on chemical contaminants in milk. *Critical Reviews in Microbiology*.

[3] Medhammar E., Wijesinha-Bettoni R., Stadlmayr B., Nilsson E., Charrondiere U. R., Burlingame B. (2012). Composition of milk from minor dairy animals and buffalo breeds: A biodiversity perspective. *Journal of the Science of Food and Agriculture*.

[4] Aspri M., Economou N., Papademas P. (2017). Donkey milk: An overview on functionality, technology, and future prospects. *Food Reviews International*.

[5] Iacono G., Carroccio A., Cavataio F., Montalto G., Soresi M., Balsamo V. (1992). Use of ass’ milk in multiple food allergy. *Journal of Pediatric Gastroenterology and Nutrition*.

[6] Carroccio A., Cavataio F., Montalto G., D'Amico D., Alabrese L., Iacono G. (2000). Intolerance to hydrolysed cow's milk proteins in infants: Clinical characteristics and dietary treatment. *Clinical and Experimental Allergy*.

[7] Monti G., Bertino E., Muratore M. C. (2007). Efficacy of donkey's milk in treating highly problematic cow's milk allergic children: An in vivo and in vitro study. *Pediatric Allergy and Immunology*.

[8] Chiofalo B., Salimei E., Chiofalo L. (2003). Acidi grassi nel latte di asina: proprietà bionutrizionali ed extranutrizionali. *Large Animals Review*.

[9] Polidori P., Vincenzetti S. Quantificazione del lisozima nel latte di asina in fasi diverse della lattazione.

[10] Tafaro A., Magrone T., Jirillo F. (2007). Immunological properties of donkey's milk: Its potential use in the prevention of atherosclerosis. *Current Pharmaceutical Design*.

[11] Perna A., Intaglietta I., Simonetti A., Gambacorta E. (2015). Donkey milk for manufacture of novel functional fermented beverages. *Journal of Food Science*.

[12] Giacometti F., Bardasi L., Merialdi G. (2016). Shelf life of donkey milk subjected to different treatment and storage conditions. *Journal of Dairy Science*.

[13] Martini M., Salari F., Altomonte I. (2018). Effects of pasteurization and storage conditions on donkey milk nutritional and hygienic characteristics. *Journal of Dairy Research*.

[14] Conte F., Scatassa M. L., Todaro M., Barreca M. (2005). Osservazioni su alcuni parametri di composizione e igienico-sanitari del latte d'asina. *Industrie Alimentari*.

[15] Verraes C., Claeys W., Cardoen S. (2014). A review of the microbiological hazards of raw milk from animal species other than cows. *International Dairy Journal*.

[16] Mancianti F., Nardoni S., Papini R. (2014). Detection and genotyping of *Toxoplasma gondii* DNA in the blood and milk of naturally infected donkeys (*Equus asinus*). *Parsites and Vectors*.

[17] Anon (2004). Regulation (EC) No 852/2004 of the European Parliament and of the Council of 29 April 2004 on the hygiene of foodstuff. *Official Journal of the European Union*.

[18] Anon (2002). Regulation (EC) No 178/2002 of the European Parliament and of the Council of 28 January 2002 laying down the general principles and requirements of food law, establishing the European Food Safety Authority and laying down procedures in matters of food safety. *Official Journal of the European Communities*.

[19] Anon (2017). Regulation (EU) No 625/2017 of the European Parliament and of the Council of 15 March 2017 amending Regulations (EC) No 999/2001, (EC) No 396/2005, (EC) No 1069/2009, (EC) No 1107/2009, (EU) No 1151/2012, (EU) No 652/2014, (EU) 2016/429 and (EU) 2016/2031 of the European Parliament and of the Council, Council Regulations (EC) No 1/2005 and (EC) No 1099/2009 and Council Directives 98/58/EC, 1999/74/EC, 2007/43/EC, 2008/119/EC and 2008/120/EC, and repealing Regulations (EC) No 854/2004 and (EC) No 882/2004 of the European Parliament and of the Council, Council Directives 89/608/EEC, 89/662/EEC, 90/425/EEC, 91/496/EEC, 96/23/EC, 96/93/EC and 97/78/ EC and Council Decision 92/438/EEC (Official Controls Regulation). *Official Journal of the European Union*.

[20] Zastempowska E., Grajewski J., Twarużek M. (2016). Food-borne pathogens and contaminants in raw milk – a review. *Annals of Animal Science*.

[21] Oliver S. P., Boor K. J., Murphy S. C., Murinda S. E. (2009). Food safety hazards associated with consumption of raw milk. *Foodborne Pathogens and Disease*.

[22] Conte F. (2014). La filiera lattiero-casearia. *Igiene Degli Alimenti. Aspetti Igienico-Sanitari*.

[23] Conte F., Passantino A. (2008). Isolation of *Enterobacter sakazakii* from ass' milk in Sicily: Case report, safety and legal issues. *Travel Medicine and Infectious Disease*.

[24] Pilla R., Daprà V., Zecconi A., Piccinini R. (2010). Hygienic and health characteristics of donkey milk during a follow-up study. *Journal of Dairy Research*.

[25] Scatassa M., Carrozzo A., Ducato B. (2011). *Bacillus cereus*: isolation in jennet milk. *Italian Journal of Food Safety*.

[26] Giribaldi M., Antoniazzi S., Gariglio G. M., Coscia A., Bertino E., Cavallarin L. (2017). A preliminary assessment of HTST processing on donkey milk. *Veterinary Sciences*.

[27] Alberghini L., Catellani P., Norbiato M. A., Giaccone V. (2012). Indagine preliminare sulle caratteristiche microbiologiche del latte dasina. *Italian Journal of Food Safety*.

[28] Momtaz H., Farzan R., Rahimi E., Dehkordi F. S., Souod N. (2012). Molecular characterization of shiga toxin-producing *Escherichia coli* isolated from ruminant and donkey raw milk samples and traditional dairy products in Iran. *The Scientific World Journal*.

[29] Mottola A., Alberghini L., Giaccone V., Marchetti P., Tantillo G., Di Pinto A. (2018). Microbiological safety and quality of Italian donkey milk. *Journal of Food Safety*.

[30] Sarno E., Santoro A. M. L., Di Palo R., Costanzo N. (2012). Microbiological quality of raw donkey milk from Campania region. * Italian Journal of Animal Science*.

[31] van Asselt E. D., van der Fels-Klerx H. J., Marvin H. J. P., van Bokhorst-van de Veen H., Groot M. N. (2017). Overview of food safety hazards in the european dairy supply chain. *Comprehensive Reviews in Food Science and Food Safety*.

[32] Conte F., Mazzullo G., Lo Verde V. (2003). Mastite nell'asina: descrizione di tre casi in Sicilia. Valutazioni qualitative sul latte, reperti citologici e considerazioni igienico-sanitarie. *Large Animals Review*.

[33] Ragona G., Corrias F., Benedetti M. (2016). Amiata donkey milk chain: Animal health evaluation and milk quality. *Italian Journal of Food Safety*.

[34] EFSA BIOHAZ Panel (EFSA Panel on Biological Hazards) (2015). Scientific opinion on the public health risks related to the consumption of raw drinking milk. *EFSA Journal*.

[35] Naccari F., Foti M., Giacopello C. (2009). Methicillin resistant *Staphylococcus* sp isolated from donkeys milk in Sicily: preliminary study. *Journal of Veterinary Pharmacology and Terapeutic*.

[36] Gul S. T., Khan A. (2007). Epidemiology and epizootology of brucellosis: a review. *Pakistan Veterinary Journal*.

[37] Acosta-González R. I., González-Reyes I., Flores-Gutiérrez G. H. (2006). Prevalence of *Brucella abortus* antibodies in equines of a tropical region of Mexico. *Canadian Journal of Veterinary Research*.

[38] Abdalla M. A. E., Abdalla S. H., Elzaki R. (2010). Prevalence of *Brucella abortus* antibodies in donkeys in Gaderef State of Eastern Sudan. *World Food System - A Contribution from Europe*.

[39] Sadiq M. A., Tijjani A.-N., Auwal M. S., Mustapha A. R., Gulani I. (2013). Serological prevalence of brucellosis among donkeys (*Equus asinus*) in some local government areas of Yobe State, Nigeria. *Journal of Equine Veterinary Science*.

[40] Ali M., Baber M., Hussain T., Awan F., Nadeem A. (2014). The contribution of donkeys to human health. *Equine Veterinary Journal*.

[41] Pavlík I., Jahn P., Dvorska L., Bartos M., Novotny L., Halouzka R. (2004). Mycobacterial infections in horses: a review of the literature. *Veterinarni Medicina*.

[42] Lyashchenko K. P., Greenwald R., Esfandiari J. (2012). Pulmonary disease due to *Mycobacterium tuberculosis* in a horse: zoonotic concerns and limitations of antemortem testing. *Veterinary Medicine International*.

[43] Boughattas S. (2017). Toxoplasma infection and milk consumption: Meta-analysis of assumptions and evidences. *Critical Reviews in Food Science and Nutrition*.

[44] Tenter A. M., Heckeroth A. R., Weiss L. M. (2000). *Toxoplasma gondii*: from animals to humans. *International Journal for Parasitology*.

[45] Haridy F. M., Saleh N. M. K., Khalil H. H. M., Morsy T. A. (2010). Anti-*Toxoplasma gondii* antibodies in working donkeys and donkey's milk in greater Cairo, Egypt. *Journal of the Egyptian Society of Parasitology*.

[46] Martini M., Altomonte I., Mancianti F., Nardoni S., Mugnaini L., Salari F. (2014). A preliminary study on the quality and safety of milk in donkeys positive for *Toxoplasma gondii*. *Animal*.

[47] Laatamna A. E., Wagnerová P., Sak B. (2015). Microsporidia and Cryptosporidium in horses and donkeys in Algeria: Detection of a novel *Cryptosporidium hominis* subtype family (Ik) in a horse. *Veterinary Parasitology*.

[48] Bornay-Llinares F. J., Da Silva A. J., Moura H. (1998). Immunologic, microscopic, and molecular evidence of *Encephalitozoon intestinalis* (*Septata intestinalis*) infection in mammals other than humans. *The Journal of Infectious Diseases*.

[49] Yue D., Ma J., Li F. (2017). Occurrence of *Enterocytozoon bieneusi* in donkeys (*Equus asinus*) in China: a public health concern. *Frontiers in Microbiology*.

[50] Lallo M. A., Vidoto Da Costa L. F., Alvares-Saraiva A. M. (2016). Culture and propagation of microsporidia of veterinary interest. *Journal of Veterinary Medical Science*.

[51] Santn M., Cortés Vecino J. A., Fayer R. (2010). A zoonotic genotype of *Enterocytozoon bieneusi* in horses. *Journal of Parasitology*.

[52] Veronesi F., Passamonti F., Cacciò S., Diaferia M., Piergili Fioretti D. (2010). Epidemiological survey on equine Cryptosporidium and Giardia infections in Italy and molecular characterization of isolates. *Zoonoses and Public Health*.

[53] Zhang X.-X., Zhang F.-K., Li F.-C. (2017). The presence of *Giardia intestinalis* in donkeys, *Equus asinus*, in China. *Parasite and Vectors*.

[54] Rieille N., Bressanelli S., Freire C. C. M. (2014). Prevalence and phylogenetic analysis of tick-borne encephalitis virus (TBEV) in field-collected ticks (*Ixodes ricinus*) in southern Switzerland. *Parasites and Vectors*.

[55] Rieille N., Klaus C., Hoffmann D., Péter O., Voordouw M. J. (2017). Goats as sentinel hosts for the detection of tick-borne encephalitis risk areas in the Canton of Valais, Switzerland. *BMC Veterinary Research*.

[56] Rahimi E., Doosti A., Ameri M., Kabiri E., Sharifian B. (2010). Detection of *Coxiella burnetii* by Nested PCR in bulk milk samples from dairy bovine, ovine, and caprine herds in Iran. *Zoonoses and Public Health*.

[57] Khaniki G. R. J. (2007). Chemical contaminants in milk and public health concerns: A review. *International Journal of Dairy Science*.

[58] Rather I. A., Koh W. Y., Paek W. K., Lim J. (2017). The Sources of chemical contaminants in food and their health implications. *Frontiers in Pharmacology*.

[59] Potortì A. G., Di Bella G., Lo Turco V., Rando R., Dugo G. (2013). Non-toxic and potentially toxic elements in Italian donkey milk by ICP-MS and multivariate analysis. *Journal of Food Composition and Analysis*.

[60] Anon (2006). Commission Regulation (EC) No 1881/2006 of 19 December 2006 setting maximum levels for certain contaminants in foodstuffs. *Official Journal of the European Union*.

[61] Anon (2004). Regulation (EC) No 853/2004 of the European Parliament and of the Council of 29 April 2004 laying down specific hygiene rules for food of animal origin. *Official Journal of the European Union*.

[62] Fantuz F., Ferraro S., Todini L. (2015). Minor and potentially toxic trace elements in milk and blood serum of dairy donkeys. *Journal of Dairy Science*.

[63] Passantino A., Russo C., Passantino L., Conte F. (2011). Ivermectin residues in milk of lactating donkey (*Equus asinus*): Current regulation and challenges for the future. *Journal für Verbraucherschutz und Lebensmittelsicherheit*.

[64] Paksoy N., Dinç H., Altun S. K. (2018). Evaluation of levels of essential elements and heavy metals in milks of dairy donkeys, goats and sheep in Turkey. *Pakistan Journal Of Zoology*.

[65] Di Bella G., Conte F., Fotia V., Rando R., mo Dugo G. (2010). Valutazione di POCs e PCBs in campioni siciliani di latte d'asina. *Atti del Convegno*.

[66] Di Bella G., Potortì A. G., Lo Turco V., Licata P., Rastrelli L., Dugo G. (2014). Donkey's milk safety: POCs and PCBs levels and infant daily intake. *Food Control*.

[67] Bilandžić N., Božić D., Dokić M. (2014). Assessment of aflatoxin M1 contamination in the milk of four dairy species in Croatia. *Food Control*.

[68] Galvano F., Galofaro V., Ritieni A., Bognanno M., De Angelis A., Galvano G. (2001). Survey of the occurrence of aflatoxin M1 in dairy products marketed in Italy: Second year of observation. *Food Additives and Contaminants: Part A*.

[69] Iqbal S. Z., Jinap S., Pirouz A. A., Ahmad Faizal A. R. (2015). Aflatoxin M1 in milk and dairy products, occurrence and recent challenges: A review. *Trends in Food Science and Technology*.

[70] Cascone G., Tumino G., Antoci F., Salina F., Lo Magno G., Milonis E., Polidori P. (2011). Qualità igienico-sanitaria del latte d’asina: esperienza in un allevamento di asine di razza Ragusana. *LATTE DI ASINA.Produzione, Caratteristiche e Gestione Dell’Azienda Asinina*.

[71] Kos J., Lević J., Duragić O., Kokić B., Miladinović I. (2014). Occurrence and estimation of aflatoxin M1 exposure in milk in Serbia. *Food Control*.

[72] Malissiova E., Manouras A. (2017). Monitoring aflatoxin M1 levels in donkey milk produced in Greece, intended for human consumption. *World Mycotoxin Journal*.

[73] Gokbulut C., Naturali S., Rufrano D., Anastasio A., Yalinkilinc H. S., Veneziano V. (2013). Plasma disposition and milk excretion of eprinomectin following pour-on administration in lactating donkeys. *Journal of Veterinary Pharmacology and Therapeutics*.

[74] Grosenbaugh D. A., Reinemeyer C. R., Figueiredo M. D. (2011). Pharmacology and therapeutics in donkeys. *Equine Veterinary Education*.

[75] Anon (2014). Commission Implementing Regulation (EU) No 418/2014 of 24 April 2014 amending the Annex to Regulation (EU) No 37/2010 on pharmacologically active substances and their classification regarding maximum residue limits in foodstuffs of animal origin, as regards the substance ivermectin. *Official Journal of the European Union*.

[76] Escribano M., San Andrés M. I., de Lucas J. J., González-Canga A. (2012). Ivermectin residue depletion in food producing species and its presence in animal foodstuffs with a view to human safety. *Current Pharmaceutical Biotechnology*.

[77] Conte F., Minniti A., Scatassa M. L., Mons G., Monsù G. (2002). Indagine preliminare sulla qualità merceologica ed igienico-sanitaria del latte di asine allevate in Sicilia. *Atti della Società Italiana delle Scienze Veterinarie*.

[78] Conte F., Scatassa M. L., Monsù G. (2003). Rilievi su composizione e qualità igienico – sanitaria del latte di asine allevate in Sicilia. *Atti del Convegno Nazionale A.I.V.I*.

[79] Zhang X.-Y., Zhao L., Jiang L., Dong M.-L., Ren F.-Z. (2008). The antimicrobial activity of donkey milk and its microflora changes during storage. *Food Control*.

[80] Colavita G., Amadoro C., Maglieri C., Sorrentino E., Varisco G., Salimei E. Hygiene and health parameters of donkeys milk.

[81] Salerno M., Paterlini F., Martino P. A., Milonis E., Polidori P. (2011). Microbiologia e attvità battericida del latte di asina. *Latte di Asina. Produzione, Caratteristiche e Gestione Dell'Azienda Asinina*.

[82] Conte F., Foti M., Malvisi M. C., Giacopello C., Piccinini R. (2012). Valutazione dell'azione antibatterica del lisozima del latte dasina. Considerazioni igienico sanitarie. *Large Animals Review*.

[83] Cavallarin L., Giribaldi M., Soto-Del Rio M. D. L. D. (2015). A survey on the milk chemical and microbiological quality in dairy donkey farms located in NorthWestern Italy. *Food Control*.

[84] Malissiova E., Arsenos G., Papademas P. (2016). Assessment of donkey milk chemical, microbiological and sensory attributes in Greece and Cyprus. *International Journal of Dairy Technology*.

[85] Di Bella G., Lo Turco V., Potortì A. G. (2012). Classification of the geographical origin of Italian donkey's milk based on differences in inorganic anions. *Food Additives and Contaminants - Part A Chemistry, Analysis, Control, Exposure and Risk Assessment*.

